# New records of *Benthesicymus* Bate, 1881 (Dendrobranchiata, Penaeoidea, Benthesicymidae) from the abyssal depths of Taiwan

**DOI:** 10.3897/zookeys.838.33051

**Published:** 2019-04-11

**Authors:** Chien-Hui Yang, Tin-Yam Chan

**Affiliations:** 1 Center of Excellence for the Oceans, National Taiwan Ocean University, Keelung 20224, Taiwan National Taiwan Ocean University Keelung Taiwan; 2 Institute of Marine Biology, National Taiwan Ocean University, Keelung 20224, Taiwan National Taiwan Ocean University Keelung Taiwan

**Keywords:** deep-sea, new records, West Pacific

## Abstract

The deep-sea *Benthesicymus* shrimps generally inhabit waters deeper than 1000 m deep. Recent deep-sea cruises off Taiwan collected two species of *Benthesicymus* Bate, 1881 from the abyssal depths greater than 3,000 m. They are *B.crenatus* Bate, 1881 and *B.laciniatus* Rathbun, 1906. Both of them are new records for Taiwan, with *B.crenatus* also representing the deepest (5,314 m) marine animal so far known for the island. The major distinguishing characters of these two species are described and illustrated.

## Introduction

The eastern and southern coasts of Taiwan are deep-sea areas including the abyssal zone. Knowledge on the deep-sea fauna of Taiwan, however, was rather limited only until recently. The ongoing Taiwan deep-sea cruises begun in 2000 have successfully sampled the deep-sea benthic fauna off the island and reported on several abyssal decapod crustaceans (Osawa et al. 2006, 2008a, b, Baba et al. 2009, Komai and Chan 2009).

Shrimps of the genus *Benthesicymus* Bate, 1881 are generally distributed in waters between 1000 and 2000 m depth (Crosnier 1986, Kikuchi and Nemoto 1991). Like other members of the family Benthesicymidae Wood-Mason & Alcock, 1891, they have a thin integument and short rostrum. The characteristics of this genus are the presence of podobranchia on the second maxilliped to third pereiopod, a telson with an acute tip and bearing at least three pairs of movable lateral spines, and dactyli of the fourth and fifth pereiopods not being subdivided (Kikuchi and Nemoto 1991, Pérez Farfante and Kensley 1997). Only one species of *Benthesicymus*, *B.investigatoris* Alcock & Anderson, 1899, has been listed from Taiwan but without further information (Lee et al. 1999). During recent deep-sea cruises off Taiwan, many specimens of *Benthesicymus* were collected and some of them were obtained from abyssal depths of more than 3000 m, including the deepest trawling station down to 5,314 m. Careful examination of the abyssal material of *Benthesicymus* revealed two species, namely *B.crenatus* Bate, 1881 and *B.laciniatus* Rathbun, 1906. Both are new records for Taiwan and with specimens of *B.crenatus* representing the deepest marine animals currently known in Taiwan. The present work reports upon these findings.

The specimens are deposited at the National Taiwan Ocean University (NTOU). The measurement given is carapace length (cl) measured dorsally from the postorbital margin to the posterior margin of the carapace. The synonymy provided is restricted to important works on the species, and the description given is based on the material from Taiwan.

## Taxonomy

### Family Benthesicymidae Wood-Mason & Alcock, 1891

#### Genus *Benthesicymus* Bate, 1881

##### Benthesicymus crenatus

Taxon classificationAnimaliaDecapodaBenthesicymidae

Bate, 1881

Figs 1 , 2

Benthesicymus crenatus Bate, 1881: 190 (type localities: central Pacific near Low Archipelago); Bate 1888: 329, pls. 54–55; Crosnier 1986: 851, figs. 6d-e, 7d-e, 8f-g; Kikuchi and Nemoto 1991: 67, figs. 2–3; Kim et al. 2000: 7, figs 2f, g, 8c.

###### Material examined.

“TAIWAN 2005”, stn OCP296, 22°15.08'N, 121°55.09'E, 4430–4455 m, 10 Aug 2005, 2 females cl 29.2–37.3 mm (NTOU M02182). “TAIWAN 2008”, stn CP413, 22°15.06'N, 121°54.98'E, 4412–4446 m, 12 Jun 2008, 2 males cl 34.2–53.7 mm (NTOU M02183); stn CP414, 22°37.91'N, 122°32.72'E, 5011–4990 m, 13 Jun 2008, 3 males cl 25.2–37.7 mm, 1 female cl 20.2 mm (NTOU M02184); stn CP415, 22°26.16'N, 122°21.10'E, 4813–4807 m, 14 Jun 2008, 3 males cl 25.8–48.6 mm (NTOU M02185); stn CP416, 22°26.44'N, 122°21.18'E, 4824–4807 m, 15 Jun 2008, 1 male cl 25.7 mm, 1 female cl 27.1 mm (NTOU M02186). “TAIWAN 2012”, stn CP465, 22°37.56'N, 122°32.23'E, 5004–4996 m, 01 Jul 2012, 1 male cl 40.8 mm (NTOU M02187); stn CP466, 22°47.86'N, 122°29.72'E, 5226–5314 m, 02 Jun 2012, 2 males cl 28.1–33.3 mm, 3 females cl 20.1–37.3 mm (NTOU M02188); stn CP467, 22°48.01'N, 122°29.69'E, 5227–5154 m, 02 Jun 2012, 4 males cl 23.8–30.7 mm, 4 females cl 13.7–39.1 mm (NTOU M02189).

###### Description.

Integument membranous and soft. Rostrum dorsally compressed and slightly elevated into a low crest, dorsal margin with three or rarely four (only in one specimen of the present material) teeth, ventral margin without teeth. Antennal spine minute but distinct. Hepatic spine present with deep cervical groove behind it. Hepatic and branchiocardiac carinae elevated (Fig. 2A). Fourth to sixth abdominal somites with posteromedian spines. Posterior margin of fourth abdominal tergite distinctly crenate; with 19–33 (usually 23–28) teeth, medial teeth larger but obtuse (except for median tooth which is largest and acute) while lateral teeth sometimes sharper but smaller (Fig. 2B). Telson with three pairs of movable lateral spines and one pair of terminal spines, distal pair of lateral spine adjacent to terminal spine (Fig. 2C). Third maxilliped and first pereiopod both with merus and ischium bearing a sharp distomesial spine (Figs 2D–E). Distal segment of third maxilliped with sharp spine at tip (Fig. 2F). Thelycum lacking seminal receptacles but bearing median longitudinal carinae on fifth and sixth thoracic sternites. Seventh and eight thoracic sternites with large tubercle and sharp median spine, respectively (Fig. 2G). Petasma with lateral lobe generally wide and flat, except for a long strong and narrow submarginal fold at ventrolateral lobule, another shorter subdistal fold also present on dorsolateral lobule; median lobe with distal margin minutely serrated (Fig. 2I), dorsomedian lobule strongly folded and densely covered with small hooked spines (Fig. 2H).

###### Coloration.

**Figure 1.**
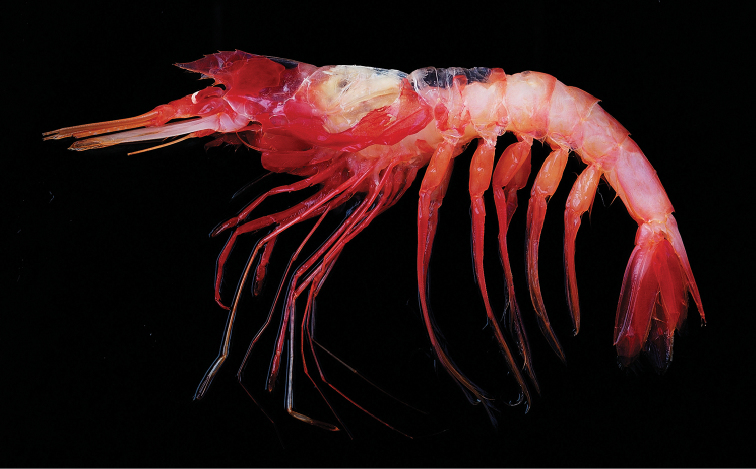
*Benthesicymuscrenatus* Bate, 1881, stn CP413, 4412–4446 m, male cl 34.2 mm (NTOU M02183).

Body entirely reddish to orangish red (Fig. 1). Antennular and antennal flagella orange. Scaphocerite and bascerite pinkish white to pale white. Cornea white.

###### Distribution.

Northwest and Central Pacific, at depths of 3,530 to 6,350 m. There is one record of 0–5,700 m for this species (Kikuchi and Nemoto 1991) but it is very likely that the material was collected from more than 3500 m deep.

###### Remarks.

The 27 specimens examined were collected from 4,412 to 5,134 m deep and most of them are damaged due to their fragile bodies even though their sizes are quite large. Nevertheless, they can be positively identified as *B.crenatus* by the characteristic comb-like crenation on the posterior margin of the fourth abdominal tergite. The Taiwanese material also generally fits well with previous descriptions of *B.crenatus* (Bate 1881, 1888, Crosnier 1986, Kikuchi and Nemoto 1991, Kim et al. 2000). The present report of *B.crenatus* from 5,324 m represents the deepest marine animal recorded from Taiwan. The previous deepest records from Taiwanese waters were the squat lobsters (Galatheidae Samouelle, 1819), *Munidopsisprofunda* Baba, 2005 and *M.taiwanica* Osawa, Lin & Chan, 2008b from 5,011 m deep (Osawa et al. 2008b, Baba et al. 2009).

The 15 species known in *Benthesicymus* (De Grave and Fransen 2011) are often separated into two groups by the following characteristics: (1) branchiostegal spine not sharp and located at margin of carapace in group I vs. very sharp but situated behind the carapace margin in group II; (2) exopod of first maxilliped abruptly narrow to tip in group I vs. tapering to tip in group II; (3) merus of second maxilliped expanded in group I vs. unexpanded in group II; (4) dactylus of third maxilliped triangular with only one terminal spine in group I vs. subrectangular with more than one spine in group II; (5) exopods of all pereiopods small but visible in group I vs. very tiny in group II (Burkenroad 1936, Kikuchi and Nemoto 1991). Groups I and II consist of ten and five species respectively, and *B.crenatus* belongs in group I.

**Figure 2.**
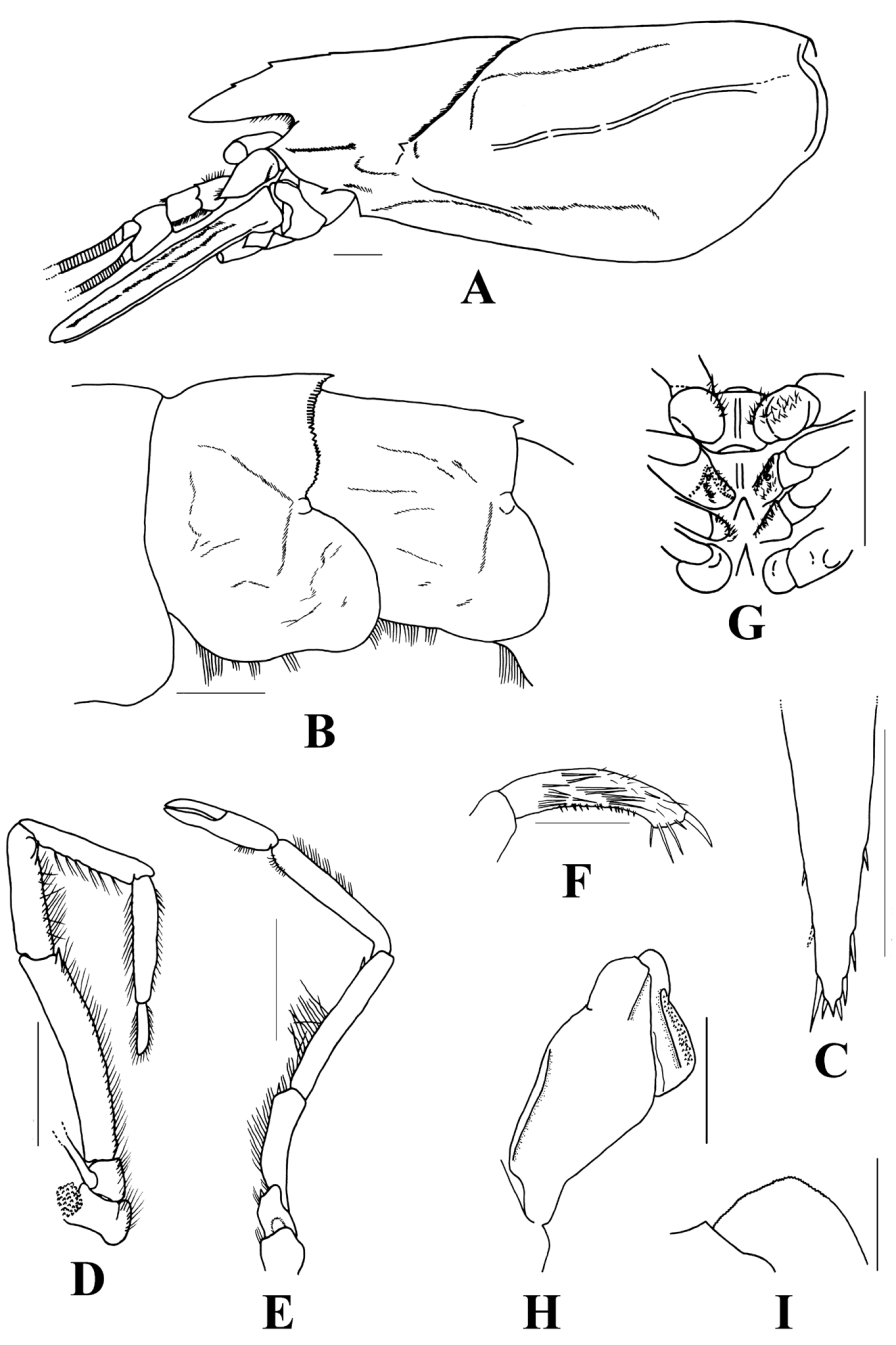
*Benthesicymuscrenatus* Bate, 1881, **A, B, H, I** stn CP413, 4412–4446 m, male cl 53.7 mm (NTOU M02183) **C** stn CP466, 5226–5314 m, female cl 20.1 mm (NTOU M02188) **D–G** stn CP416, 4824–4807 m, female cl 27.1 mm (NTOU M02186) **A** carapace and anterior appendages, lateral view **B** abdominal somites III to V, lateral view **C** telson, dorsal view **D** left maxilliped III **E** left pereiopod I **F** dactylus of left maxilliped III **G** thoracic sternites V to VIII **H** left petasma, ventral view **I** tip of median lobe, ventral view. Scale bars: 5 mm (**A–E, G, H**); 1 mm (**F, I**).

**Figure 3.**
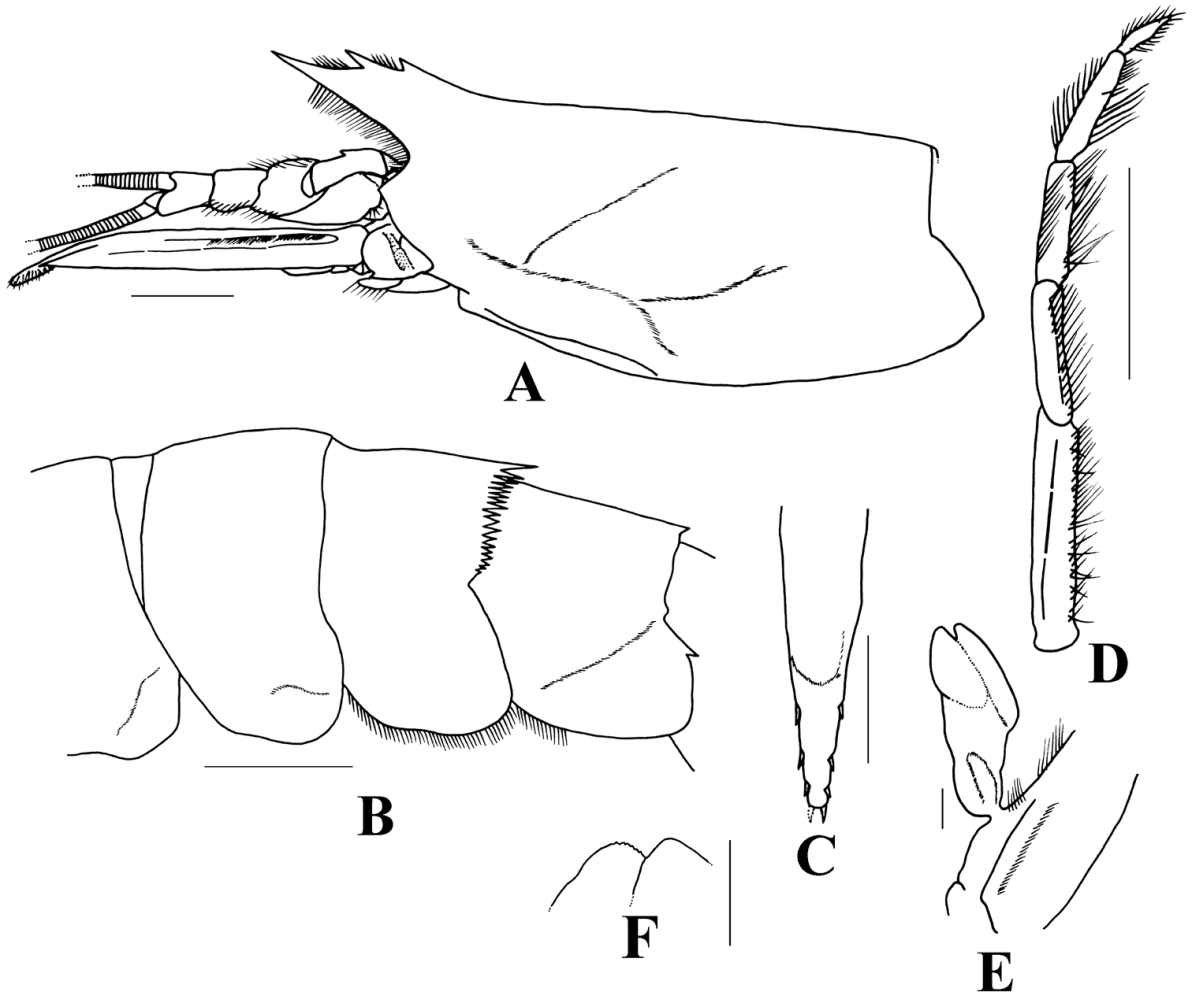
*Benthesicymuslaciniatus* Rathbun, 1906, stn CP369, 3030–3070 m, male cl 25.0 mm (NTOU M02191) **A** carapace and anterior appendages, lateral view **B** abdominal somites IV to V, lateral view **C** telson, dorsal view **D** left maxilliped III **E** right petasma, ventral view **F** tip of median lobe, ventral view. Scale bars: 5 mm (**A–D**); 1 mm (**E, F**).

The closest species to *B.crenatus* is *B.laciniatus* (Crosnier 1986, Kikuchi and Nemoto 1991), which was also collected from Taiwan (see below). The two species can be readily separated by the following characters: (1) dorsal margin of rostrum armed with three or four teeth in *B.crenatus* (Fig. 2A) but with only two teeth in *B.laciniatus* (Fig. 3A); (2) hepatic spine present in *B.crenatus* (Fig. 2A) whereas absent in *B.laciniatus* (Fig. 3A); (3) cervical groove deep and with elevated branchiocardiac carina extending to posterior carapace in *B.crenatus* (Fig. 2A), carapace without deep groove nor distinct carina in *B.laciniatus* (Fig. 3A); (4) teeth on crenation of fourth abdominal pleuron more numerous (25–29 teeth) and rather blunt in *B.crenatus* (Fig. 2B), whereas fewer (13–19 teeth) and sharp in *B.laciniatus* (Fig. 3B); (6) posterior margin of fifth abdominal pleuron bearing distinct spine in *B.laciniatus* (Fig. 3B) whereas smooth in *B.crenatus* (Fig. 2B); (7) mesial margin of merus and ischium in third maxilliped and first pereiopod with a sharp spine in *B.crenatus* (Fig. 2D–E), whereas without spine in *B.laciniatus* (Fig. 3D); (8) thelycum with strong median spine on eighth thoracic sternite in *B.crenatus* (Fig. 2G), whereas without spine in *B.laciniatus* (Crosnier 1986: fig. 6c).

##### Benthesicymus laciniatus

Taxon classificationAnimaliaDecapodaBenthesicymidae

Rathbun, 1906

Fig. 3

Benthesicymus laciniatus Rathbun 1906: 906, fig. 59, pl. 19 fig. 3 (type locality: vicinity of Kauai Island, Hawaii); Burkenroad 1936: 26, fig. 1; Crosnier 1986: 851, figs. 6c, 7a-c, 8a-e; Kikuchi and Nemoto 1991: 65.
*Benthesicymus Hjorti*Sund 1920: 30, fig. 48, pl. 11 fig. 4 (type locality: south of Canary Islands).
Gennadas pectinatus Schmitt 1921: 25, fig. 12, pl. 11 fig. 1 (type locality: off Santa Catalina Island, California).

###### Material examined.

Taiwan, “TAIWAN 2006”, stn CP366, 22°02.87'N, 121°10.08'E, 1302–1301 m, 24 Aug 2006, 1 male cl 14.3 mm (NTOU M02190); stn CP369, 24°18.96'N, 122°04.20'E, 3030–3070 m, 25 Aug 2006, 1 male cl 25.0 mm (NTOU M02191).

###### Description.

Integument moderately rigid. Rostrum rather straight, armed with two dorsal teeth. Carapace with surface rather smooth, lacking hepatic spine and without distinct grooves or carinae (Fig. 3A). Abdomen with fourth to sixth somites each armed with a distinct posteromedian spine; fourth tergite also with posterior margin distinctly crenate or serrated, bearing 17–19 sharp teeth that progressively become smaller laterally (Fig. 3B). Telson with three pairs of movable lateral spines and one pair of terminal spines, distalmost pair of lateral spines at some distance from terminal spines (Fig. 3C). No spine on both merus and ischium of third maxilliped and first pereiopod. Third maxilliped heavily setose and distal segment lacking spine (Fig. 3D). Petasma generally flat and simple, bilobed (Fig. 3E), distal margin of median lobe slightly serrated (Fig. 3F).

###### Coloration.

Unknown.

###### Distribution.

Worldwide distribution and reported from eastern Atlantic, eastern Pacific and Indo-West Pacific, at depths of approximately 1,325–4,000 m but generally from 1,500–3,000 m (Crosnier 1986).

###### Remarks.

*Benthesicymuslaciniatus* is recorded from Taiwan for the first time. As mentioned by Kim et al. (2000), *B.laciniatus* generally inhabits shallower waters than *B.crenatus* and only sometimes occurs in the abyssal zone. The two Taiwanese specimens have been collected from depths of 3,030–3,070 m and 1,301–1,302 m respectively. In *Benthesicymus* only two species have the posterior margin of the fourth abdominal tergite crenate (see Burkenroad 1936, Crosnier 1986). The distinguishing characters of these two species are given in the “Remarks” of *B.crenatus*.

The present two males collected from Taiwan generally agree with previous descriptions of the species except for the petasma with the distal margin of the median lobe not distinctly serrated (see Burkenroad 1936: fig. 1, Crosnier 1986: fig. 8a, b, e). Such difference may be due to the present males are much smaller (cl 14.3–25.0 mm vs. cl 33–36 mm for Burkenroad 1936: fig. 1; Crosnier 1986: fig. 8a, b, e) and probably juveniles.

## Supplementary Material

XML Treatment for Benthesicymus crenatus

XML Treatment for Benthesicymus laciniatus
